# Pharmacokinetics, efficacy, and safety of a novel aripiprazole microsphere-based long-acting injectable formulation for schizophrenia: A multicenter, randomized controlled trial

**DOI:** 10.1016/j.jpha.2025.101350

**Published:** 2025-05-22

**Authors:** An-Ning Li, Sheng-Chun Jin, De-Wei Shang, Jian-Xiong Guo, Hua-Li Lin, Ming Zhang, Bo Wei, Feng Wan, Yun-Long Tan, Li-Li Wang, Jian-Chu Zhou, Ping Liu, Lian-Lian Fan, Ju-Shui Sun, Bin Chen, Yimin Cui, Gang Wang

**Affiliations:** a National Clinical Research Center for Mental Disorders & National Center for Mental Disorders Beijing Anding Hospital Affiliated to Capital Medical University, Beijing, 100088, China; b Early Intervention Department, The Fourth People's Hospital of Hefei, Hefei, 230022, China; c Drug Clinical Trial Institution, The Brain Hospital Affiliated to Guangzhou Medical University, Guangzhou, 510370, China; d Medical Affairs Department, The Brain Hospital Affiliated to Guangzhou Medical University, Guangzhou, 510370, China; e Women's Psychopsychology Department, Xi'an Mental Health Center, Xi'an, 710061, China; f Psychiatric Department 6, Shandong Daizhuang Hospital, Jining, Shandong, 272000, China; g Jiangxi Provincial Psychiatric Hospital, Nanchang, 220029, China; h Female Acute Care Ward 3, Shenzhen Kangning Hospital, Shenzhen, Guangdong, 518118, China; i Beijing Huilongguan Hospital, Beijing, 100096, China; j Psychiatric Department 8, Tianjin Anding Hospital, Tianjin, 300222, China; k The 11th People's Hospital of Chongqing, Chongqing, 400030, China; l Psychosomatic Medicine Department, Deyang People's Hospital, Deyang, Sichuan, 618000, China; m Drug Clinical Trial Institution, Deyang People's Hospital, Deyang, Sichuan, 618000, China; n Premium Psychiatric Services Department, The Third People's Hospital of Huzhou, Huzhou, Zhejiang, 313000, China; o Zhuhai Livzon Microsphere Technology Co., Ltd., Livzon Pharmaceutical Group Inc., Zhuhai, Guangdong, 519090, China; p Institute of Clinical Pharmacology, Peking University First Hospital, Beijing, 100191, China

## Abstract

• A microsphere-based formulation of aripiprazole (MS350) was investigated.
• MS350 was bioequivalent to microcrystalline formulation with more stable plasma levels.
• MS350 had fewer adverse events, demonstrating a better safety profile.

## Graphical abstract

**Figure:**
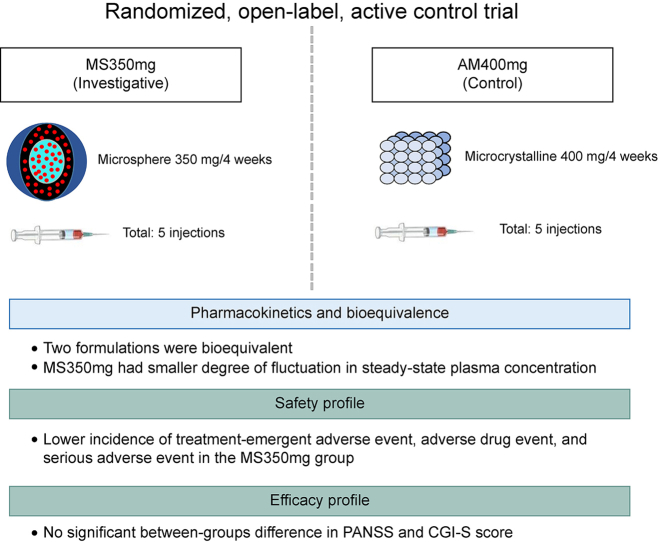

Schizophrenia is a severe and chronic psychiatric disorder with a lifetime prevalence of approximately 0.7%–1% worldwide [1]. Aripiprazole is widely used for schizophrenia treatment, and known as a dopamine system stabilizer due to its partial agonist activity as the dopamine-2 (D2) and serotonin 5-hydroxytryptamine 1A (5-HT1A) receptors, as well as antagonist action at the 5-HT2A receptors [2]. The investigational microsphere-based aripiprazole injection in this study is a novel long-acting formulation designed to optimize the release profile at the dose of 350 mg monthly. The objective of this study was to evaluate the pharmacokinetics, efficacy, and safety of the microsphere-based formulation, particularly the fluctuations in the peak-to-trough plasma concentration ratio.

The study was a multicenter, open-label, randomized, active-controlled clinical trial conducted in 16 centers in China, and enrolled a total of 113 male and 93 female eligible participants who were randomly assigned to MS350mg (investigational medicine product, aripiprazole microsphere for injection, 350 mg every four weeks; Livzon Pharmaceutical Group Inc., Zhuhai, China) or AM400mg (reference product, Abilify Maintena®, 400 mg every four weeks; Otsuka Pharmaceutical Co., Ltd., Tokyo, Japan). The inclusion and exclusion criteria are shown in Supplementary data. Based on the intention-to-treat (ITT) population, the groups were comparable in terms of baseline characteristics (Table S1). All patients received five intramuscular injections on days 1, 29, 57, 85, and 113, and a total of 29 blood samples from each patient were collected via the antecubital vein according to the following schedule: on the first injection day (day 1), samples were taken 1 h before dosing, and at 4 h and 12 h after dosing, as well as on days 2, 4, 6, 8, 12, 15, and 22. For the second to fourth injections (days 29, 57, and 85), blood samples were collected within 1 h before dosing and on day 14 post-dosing. For the fifth injection (day 113), blood samples were collected 1 h before dosing and at 4 and 12 h after dosing, as well as 2, 4, 6, 8, 12, 15, 22, 29, 43, and 57 days after last injection. The entire study lasted for 169 days from the first administration to the end of the treatment.

Pharmacokinetic parameters (Table S2) of aripiprazole were calculated using the non-compartmental approach, including the steady-state peak plasma concentration (*c*_max*,ss,*0–28_ _days_), steady-state trough plasma concentration (*c*_min*,ss,*0–28_ _days_), and the area-under-the-plasma-concentration-time curve from time zero to day-28 after the last injection (AUC_0–28 days,ss_). Linear regression analysis of the aripiprazole through concentrations from the third to fifth doses showed the estimated slopes (95% confidence interval (95% CI)) as −0.027 (−0.078 to 0.025) and 0.023 (−0.017 to 0.062) for the MS350mg and AM400mg groups respectively, indicating that the plasma concentrations had reached steady-state before final dose. After the last (fifth) administration, the *c*_max_ of the MS350mg group was equivalent to that of the AM400mg group (Fig. 1A and Table S2). In the MS350mg group, the percentage fluctuation and swing were lower than the AM400mg group after the first dose, while they were comparable to the AM400mg group after the last dose (Fig. 1B).

**Fig. 1:**
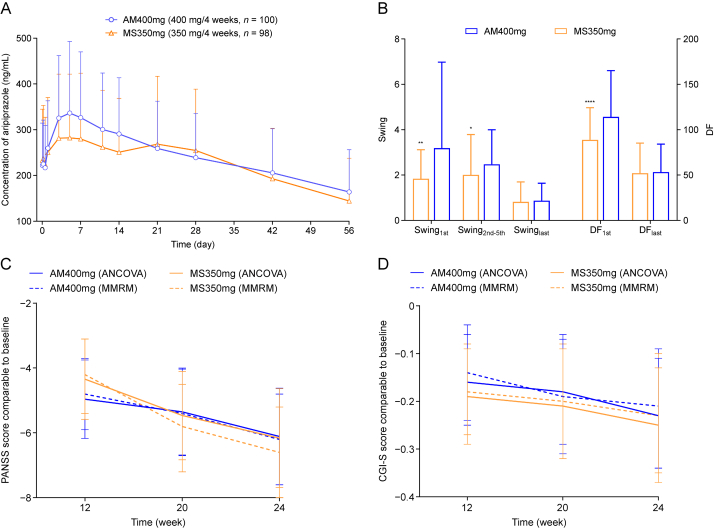
Results of the pharmacokinetics and efficacy. (A) The plasma concentration-time curve after the last injection. (B) Degree of fluctuations (DF) and swing of aripiprazole plasma concentrations through five injections. Student's *t*-test: ∗*P* < 0.05, ^∗∗^*P* < 0.01, and ^∗∗∗∗^*P* < 0.0001. (C, D) Mean changes with standard deviation (mean ± SD) in positive and negative syndrome scale (PANSS) (C) and clinical global impression-severity (CGI-S) (D) total scores at 12, 20, and 24 weeks (compared to baseline). ANCOVA: analysis of covariance; MMRM: mixed-effect model for repeated measures.

For safety analysis, adverse events (AEs) as coded according to the medical dictionary for regulatory activities (meddra) definition were recorded throughout the study, and classified by severity according to the common terminology criteria for AEs (CTCAE) (version 5.0). The incidence rates of treatment-emergent AEs (TEAEs) and adverse drug reactions (ADRs) were lower in the MS350mg group within the ITT population. Notably, the majority of TEAEs were grade 1–2 in severity, with grade 3 and above TEAEs occurring at rates of 1.0% and 4.9%, and the incidence rate where TEAEs had led to study withdrawal was 1.0% and 1.9% in the MS350mg and AM400mg group respectively. No serious AE (SAE) was reported in the MS350mg group, except for one case of grade 2–3 ADRs (weight gain). In contrast, the AM400mg group reported two SAE cases deemed unrelated to the treatment and two cases of grade 2–3 ADRs (weight gain and abnormal hepatic function). No participants withdrew from the study in the MS350mg group, while two participants from the AM400mg group withdrew (Table 1; adverse reactions with incidence rate of ≥2% are shown in Table S3). Meanwhile, the MS350mg group showed a lower incidence of extrapyramidal symptoms (EPS) related AEs (1.9%) compared to the AM400mg (6.8%), indicating a favorable safety profile for the MS350mg formulation.

**Table 1:** Number of treatment-emergent adverse events (TEAE), adverse drug events (ADR), serious adverse events (SAE), serious adverse drug event (SADR), and extrapyramidal symptoms (EPS) reported during the trial.

Events	Grade/classification	MS350mg group (*n* = 103)		AM400mg group (*n* = 103)	
		Number of participants (%)	Number of incidents	Number of participants (%)	Number of incidents
TEAE	All grades	78 (75.7)	236	86 (83.5)	284
	Grade 1	70 (68.0)	184	72 (69.9)	206
	Grade 2	32 (31.1)	51	41 (39.8)	73
	≥Grade 3	1 (1.0)	1	5 (4.9)	5
ADR	All grades	56 (54.4)	141	64 (62.1)	145
	Grade 1	53 (51.5)	119	52 (50.5)	114
	Grade 2	15 (14.6)	21	21 (20.4)	29
	≥Grade 3	1 (1.0)	1	2 (1.9)	2
SAE		0 (0)	0	2 (1.9)	2
SADR		0 (0)	0	0 (0)	0
Trial withdrawal due to	TEAE	1 (1.0)	1	2 (1.9)	2
	ADR	0 (0)	0	2 (1.9)	2
Treatment discontinuation due to	TEAE	0 (0)	0	0 (0)	0
	ADR	0 (0)	0	0 (0)	0
Death due to	SAE	0 (0)	0	0 (0)	0
	SADR	0 (0)	0	0 (0)	0
EPS	Total EPS event	2 (1.9)	5	7 (6.8)	8
	Akathisia	2 (1.9)	2	3 (2.9)	3
	Extrapyramidal disorder	0 (0)	0	3 (2.9)	3
	Tremor	0 (0)	0	2 (1.9)	2
	Drooling	1 (1.0)	1	0 (0)	0
	Musculoskeletal rigidity	1 (1.0)	2	0 (0)	0

The positive and negative syndrome scale (PANSS) total score and the clinical global impression-severity (CGI-S) score were assessed every four weeks, and differences between the two treatment groups were compared at week 12, 20, and 24. The mean changes with standard deviation (mean ± SD) in PANSS total scores from baseline at different visits for both groups are shown in Fig. 1C and Table S4. Based on the ITT population, an analysis of covariance (ANCOVA) on the changes in PANSS total score found no statistically significant differences between the treatment groups at different visits (*P*-value: 0.745, 0.909, and 0.775 respectively). Nonetheless, after adjusting for baseline covariates, the least square means (LSM) changes in PANSS total scores from baseline showed statistically significant within-group improvements from baseline in both groups. Similarly, longitudinal analysis with mixed-effect model for repeated measures (MMRM) also suggested significant differences across visits compared to baseline (*P* < 0.001), but no significant differences between groups (*P* = 0.928) or for the interaction between groups and visits (*P* = 0.393). Additionally, both groups showed consistent reduction in all PANSS subscale scores and total scores in almost all visits. Comparisons of LSM changes from baseline between the groups at the same time points revealed no statistically significant differences (*P* > 0.05), demonstrating that the efficacy of MS350mg group was comparable to that of AM400mg. Similarly, the changes of CGI-S scores in illness severity at week 12, 20, and 24 compared to baseline were significantly different for both MS350mg and AM400mg without any difference between groups (Fig. 1D and Table S5).

The PK findings of this study suggested that the fluctuation in drug concentration was significantly smaller for the MS350mg, which would produce less fluctuation in the D2-dopamine receptor occupancy to improve pharmacodynamics outcomes and reduce the risk of EPS and other AEs [3,4]. Additionally, throughout the entire study, the mean plasma concentrations of microsphere formulation remained within the therapeutic windows (94.0–534 ng/mL) [5], indicating a better control over potential burst release of drugs which led to more stable plasma levels during treatment. Therapeutic drug monitoring recommendations suggest that long-acting antipsychotics should achieve a peak-to-trough fluctuation of less than two-fold to balance efficacy and tolerability [4]. The MS350mg group not only achieved this standard but also had demonstrated smaller percentage fluctuations, fluctuation amplitudes, and peak-to-trough ratios compared to Abilify Maintena®, both after the first dose and at steady state. The observed improvement in clinical outcomes, particularly across the PANSS subscales, further corroborated the utility of MS350mg in addressing a broad spectrum of schizophrenia. All these factors may enhance patient tolerability, improve long-term treatment adherence, and be particularly suitable for patients who were more prone to experience dose-related side-effects.

This study demonstrated that the novel microsphere long-acting injection formulation is comparable to Abilify Maintena® in efficacy and safety profile, and had a more stable pharmacokinetic profile with lower degree of fluctuation (DF) that may contribute to improved tolerability and fewer dose-related side effects. In general, these findings support the microsphere formulation as a viable alternative to the microcrystalline formulation for the long-term treatment of schizophrenia patients.

## CRediT authorship contribution statement

**An-Ning Li:** Writing – review & editing, Methodology, Funding acquisition, Conceptualization. **Sheng-Chun Jin:** Writing – original draft, Visualization, Methodology, Formal analysis. **De-Wei Shang:** Writing – original draft, Software, Formal analysis. **Jian-Xiong Guo:** Investigation, Data curation. **Hua-Li Lin:** Investigation, Data curation. **Ming Zhang:** Investigation, Data curation. **Bo Wei:** Investigation, Data curation. **Feng Wan:** Investigation, Data curation. **Yun-Long Tan:** Project administration, Formal analysis. **Li-Li Wang:** Investigation, Formal analysis. **Jian-Chu Zhou:** Project administration, Formal analysis. **Ping Liu:** Supervision, Methodology, Conceptualization. **Lian-Lian Fan:** Supervision, Methodology, Conceptualization. **Ju-Shui Sun:** Supervision, Methodology, Conceptualization. **Bin Chen:** Writing – review & editing, Supervision, Funding acquisition. **Yimin Cui:** Supervision, Methodology, Funding acquisition. **Gang Wang:** Supervision, Funding acquisition.

## Ethical statement

This study was performed in compliance with a project license (Approval No.: (2021) Review (79)2121138FS-2) granted by the ethics committees of Beijing Anding Hospital Affiliated to Capital Medical University, The Brain Hospital Affiliated to Guangzhou Medical University, Beijing Huilongguan Hospital, Tianjin Anding Hospital, Xi'an Mental Health Center, Shandong Daizhuang Hospital, Wuhan Mental Health Center, The 11th People's Hospital of Chongqing, Deyang People's Hospital, The First People’s Hospital of Shanxi Medical University, The Fourth People's Hospital of Hefei, Jiangxi Provincial Psychiatric Hospital, Shenzhen Kangning Hospital, and The Third People's Hospital of Huzhou. Informed consents were obtained from all patients participating in the trial, and they signed the paper informed consent forms.

## Declaration of competing interest

The authors declare that there are no financial interests or personal relationships that may be considered as potential competing interest: author Bin Chen severd as Deputy General Manager in Livzon Pharmaceutical Group Inc. during the research. Moreover, Livzon Pharmaceutical Group Inc. has no relevant relationships or competing interests related to this research.
