# Supplementary material for: Pharmacokinetics, efficacy, and safety of a novel aripiprazole microsphere-based long-acting injectable formulation for schizophrenia: A multicenter, randomized controlled trial
Source: J Pharm Anal. 2025 May 22;16(1):101350. doi: 10.1016/j.jpha.2025.101350 (PMC12856284; doi:10.1016/j.jpha.2025.101350)
Supplement: Multimedia component 1 [file mmc1.docx]

Supplementary Material

1. Method
   1. Trial design and participants

Eligible participants were aged between 18 to 64 years old, had a body mass index (BMI) between 18.5 (inclusive) and 35.0 (exclusive) kg/m^2^ where the body weights must be at least 45 kg and 50 kg for female and male patients, respectively. Participants had a diagnosis of schizophrenia and were clinically stable at the time of enrollment, had received oral antipsychotic treatments (excluding clozapine, chlorpromazine, haloperidol, and thioridazine) for at least four weeks. Clinical stability was confirmed through assessments by qualified psychiatrists, where the Positive and Negative Syndrome Scale (PANSS) score at both the screening and baseline visits were ≤70. During the screening phase, participants were transitioned to a treatment regimen of aripiprazole tablets (Abilify®, Zhejiang Otsuka Pharmaceutical Co., Ltd., Zhejiang，China) at 10mg per day, along with no more than one other antipsychotic medication for a minimum of 14 days.

We excluded patients who had other comorbid psychiatric disorders (other than schizophrenia) as diagnosed by the Diagnostic and Statistical Manual of Mental Disorders (DSM-IV-TR) criteria. Patients with total bilirubin or creatinine levels exceeding 1.5 times the upper limit of normal (ULN), or alanine aminotransferase (ALT) or aspartate aminotransferase (AST) levels exceeding 2 times the ULN, were also excluded. Additionally, patients who had used clozapine, chlorpromazine, haloperidol, or thioridazine within 4 weeks before the first administration of the study drugs, those who had taken Cytochrome P450 proteins (CYP3A4 or CYP2D6) inhibitors or inducers, or any prescription traditional Chinese medicines within 2 weeks before the study enrollment, or those who had last received treatment with long acting aripiprazole injection (including both intervention and reference medication) within one year of the study’s initiation, were not eligible. Lastly, we also excluded patients with uncontrolled or unstable comorbidities, such as cardiovascular, endocrine, respiratory, hematological, or immunological diseases, or any other conditions deemed unsuitable for participation by the study investigators.

- 1. Trial procedures

Eligible participants were randomly assigned to one of two treatment regimens: MS350mg (aripiprazole microsphere for injection, 350 mg every 4 weeks) and AM400mg (Ability Maintena®, 400 mg every 4 weeks). The randomization sequence was generated by an independent statistician who was not involved in patient selection, recruitment, or randomization, in a block size of 10 and stratified according to CYP2D6 genotype. The randomization process was undertaken by each individual study center through a centralized system（eBalance, Taimei Medical Technology Co., Ltd, China. The researcher who perform the analysis were blinded towards patient allocation.

All the patients received five intramuscular injections on day 1, 29, 57, 85 and 113, and a total of 29 blood samples were collected via the antecubital vein according to the following schedule: on the first injection day (day 1), samples were taken 1 h before dosing, and at 4 h and 12 h after dosing, as well as on days 2, 4, 6, 8, 12, 15, and 22. For the second to fourth injections (days 29, 57, and 85), blood samples were collected within 1 h before dosing and on day 14 post-dosing. For the fifth injection (day 113), blood samples were collected 1 h before dosing and at 4 h and 12 h after dosing, as well as 2, 4, 6, 8, 12, 15, 22, 29, 43, and 57 days after last injection.

Blood samples were collected at room temperature into the EDTA-K_2_ tubes and centrifuged within 120 min of collection. The centrifugation was performed at 4℃ and 1500 g for 15 min, after which the plasma was separated, collected, and stored at -70℃ until analysis. The plasma samples were analyzed in a centralized, Good Laboratory Practice (GLP)-certified laboratory (WuXi AppTec, Shanghai, China) using a validated liquid chromatography-tandem mass spectrometry (LC-MS/MS) method to determine the concentrations of aripiprazole Deuterated aripriprazole-d8 was used as internal standards.

- 1. LC-MS/MS method

Binary Pump LC-30AD (Shimadzu) tandem API 5500 (Applied Biosystems/Sciex) mass spectrometry was used to determine the drug in plasma. Chromatographic conditions: Waters ACQUITY UPLC BEH C18 column (2.1 × 50 mm, 1.7 µm), with a mobile phase consisting of water containing 0.04% formic acid and 10 mM ammonium formate as mobile phase A, and a 95% acetonitrile-water solution as mobile phase B. The flow rate was set at 0.6 mL·min^⁻¹^, the column temperature was maintained at 50°C, and the injection volume was 3 µL. Gradient elution was performed as follows: 0–0.2 min, 30% B; 0.20–1.50 min, linear gradient from 30% to 50% B; 1.51–2.50 min, 100% B; 2.51–3.20 min, return to 30% B; 3.20–4.50 min, linear gradient from 30% to 50% B; 4.51–5.00 min, 90% B; 5.01–5.50 min, re-equilibration at 30% B.

Mass spectrometry conditions: Under ESI positive mode, Secondary mass spectrometry analysis was conducted in multiple reaction monitoring (MRM) mode. The MRM transitions used for quantification were as follows: Aripiprazole, m/z 448.1 → m/z 128.1; Aripiprazole-d8 (internal standard), m/z 456.1 → m/z 293.1. The declustering potential (DP) was 80 V, entrance potential (EP) was 10 V, dwell time was 50 ms, collision energy was 42 eV, and collision cell exit potential (CXP) was 17 V.

Sample preparation: To 50 µL of plasma sample, 20 µL of internal standard working solution was added, followed by the addition of 300 µL of acetonitrile. The mixture was vortexed and centrifuged at 6200 r·min^⁻¹^ for 10 min at 12°C. Subsequently, 200 µL of the supernatant was diluted with 100 µL of water prior to injection and detection. The calibration curve exhibited good linearity within the range of 1.50–750 ng/mL. Extraction recoveries for low, medium, and high concentration quality control samples were 90.0%, 94.2%, and 89.7%, respectively. Intra- and inter-batch accuracy of quality control samples was within ±15% (±20% for the lower limit of quantitation), while intra- and inter-batch precision did not exceed 15% (20% for the lower limit of quantitation). Residuals after high concentration samples were no more than 20% of the lower limit of quantitation and 5% of the internal standard. Samples were stable throughout the testing period.

1. Outcomes and statistical methods
   1. Pharmacokinetic Evaluation and Safety Analysis

Pharmacokinetic parameters were calculated using Phoenix WinNolin^TM^ software (version 8.3.4, Certara, Inc, USA). For safety analysis, adverse events (AEs) as coded according to the Medical Dictionary for Regulatory Activities (MedDRA) definition were recorded throughout the study, and classified by severity according to the Common Terminology Criteria for Adverse Events (CTCAE, version 5.0).

- 1. Plasma concentration fluctuations

Descriptive analysis was performed on the degree of fluctuation (DF) and the swing in plasma concentration. The resultant arithmetic and geometric means and coefficient of variation, standard deviation, quartiles, and minimum-maximum values were tabulated. Additionally, a stratified descriptive analysis was performed based on CYP2D6 phenotypes (poor, normal, intermediate, and ultra-rapid metabolizers). No inferential statistical calculation was performed. Both DF and swing for the first dose were calculated using equation (1) and (2) below:

$DF_{dose1}=\frac{C_{max,d1}-C_{d28}}{C_{average,d1}} \times100\%$ (1)

$Swing_{dose1}=\frac{C_{max,d1}-C_{d28}}{C_{d28}}$ (2)

Similar calculations of DF and Swing were performed for the last dose. For the 2nd to 5th dose, only swing was calculated as the average concentration of aripiprazole could not be calculated due to sparse sampling. Student’s t-test was used to compare the difference of DF and swing between two formulations with IBM SPSS Statistics software (version 26, IBM Corp.).

- 1. Mixed-effect model for repeated measures

The model used changes from baseline as the dependent variable (outcome), and no imputation was required. Fixed effects of the model included treatment group, follow-up visits (time point), and the interaction between treatment group and follow-up visits. Baseline measurements were included as the covariates to account for initial differences, and study center was treated as a random effect. In analyses of group differences, the AM400mg group was considered as the reference group. As the efficacy analysis was preliminary, no adjustment for type I error was applied, and all comparisons were conducted a two-sided significance level where α=0.05

Table S1: Demographic and baseline characteristics.

| Characteristics (mean±SD) | MS350mg (n=103) | AM400mg (n=103) |
| --- | --- | --- |
| Age, year | 39.1±12.41 | 39.7±12.15 |
| Weight, kg | 69.52±11.561 | 69.33±12.921 |
| BMI, kg/m^2^ | 25.33±3.61 | 25.53±3.84 |
| Waist circumference, cm | 89.73±10.04 | 90.29±11.37 |
| Male sex [number (%)] | 58 (56.3) | 55 (53.4) |
| Ethnicity [number (%)] | | |
| Han | 100 (97.1) | 101 (98.1) |
| Others | 3 (2.9) | 2 (1.9) |
| CYP2D6 genetic polymorphism [number (%)] | | |
| Poor metaboliser | 0 (0) | 0 (0) |
| Medium metaboliser | 48 (46.6) | 48 (46.6) |
| Fast metaboliser | 55 (53.4) | 54 (52.4) |
| Ultra-fast metaboliser | 0 (0) | 1 (0) |

SD, standard deviation. BMI, body mass index. CYP, Cytochrome P450.

Table S2: Steady state pharmacokinetic characteristics of aripiprazole microsphere for injection and Abilify Maintena^®^

| Parameters | MS350mg (n=103, mean±SD) | AM400mg (n=103, mean±SD) |
| --- | --- | --- |
| *t_max, ss_* (d)^a^ | 7.02 (3.00–21.00) | 5.03 (4.94–10.19) |
| *C_max, ss_* (ng/mL) | 335.90±173.92 | 355.84±154.57 |
| *C_min, ss_* (ng/mL) | 196.10±90.49 | 200.68±78.83 |
| *C_av, ss_* (ng/mL) | 263.16±122.04 | 283.19±108.03 |
| AUC_0–28d, ss_ (day⸱ng/mL) | 7368.48±3417.12 | 7929.31±3024.78 |
| AUC_0-t_ (day⸱ng/mL) | 12768.92±5724.29 | 13459.46±5164.24 |
| AUC_0-∞_(day⸱ng/mL) | 168285.36±1161231.16 | 29319.32±20528.77 |
| *V_z_/F*(L)^b^ | 2422.31 (984.44–5941.09) | 3447.29 (2301.61–6098.28) |
| *CL_ss_/F*(L/h) | 2.36±1.04 | 2.42±0.97 |
| *λ_z_* (10^-3^/h) | 1.33±1.31 | 0.73±0.46 |
| *t_1/2_* (h)^b^ | 31.99 (17.27–70.25) | 46.01 (29.93–69.89) |
| Ra (C_max_) (adim) | 1.05±0.43 | 1.01±0.50 |
| Ra (AUC) (adim) | 1.23±0.45 | 1.25±0.54 |

a: Data is shown as median (range).

b: Data is shown as mean (range).

SD: standard deviation. *T_max, ss_:* time of the steady-state peak plasma concentration. *C_max, ss_*: steady-state peak plasma concentration. *C_min, ss_*: steady-state through plasma concentration. *C_av, ss_*: steady-state average plasma concentration. AUC_0–28d, ss_: area-under-the-plasma-concentration-time curve from time zero to day-28 after the last injection. AUC_0-t_: area-under-the-plasma-concentration-time curve from time zero to the time of the last measurable (positive) concentration after the last injection. AUC_0-∞_: area-under-the-plasma-concentration-time curve from time zero extrapolated to infinity after the last injection. *V_z_/F*: volume of distribution. *CL_ss_/F*: total body clearance. *λ_z_*: First-order rate constant associated with the terminal (log-linear) portion of the curve. *t_1/2_*: terminal half-life. Ra: the ratio of the last injection to the first injection. adim: dimensionless.

Table S3: Adverse reactions with an incidence rate of ≥2% in either group

| System Organ Classification | MS350mg (n=103) | AM400mg (n=103) |
| --- | --- | --- |
|  | n (%) | n (%) |
| *Investigations* | | |
| Weight gain | 16 (15.5) | 10 (9.7) |
| Weight loss | 17 (16.5) | 11 (10.7) |
| Blood prolactin increased | 5 (4.9) | 5 (4.9) |
| Alanine aminotransferase increased | 3 (2.9) | 5 (4.9) |
| Leukocytes urine positive | 3 (2.9) | 5 (4.9) |
| Neutrophil count increased | 0 (0) | 4 (3.9) |
| Aspartate aminotransferase increased | 3 (2.9) | 1 (1.0) |
| White blood cell count increased | 0 (0) | 5 (4.9) |
| Blood triglycerides increased | 2 (1.9) | 3 (2.9) |
| Blood glucose increased | 1 (1.0) | 3 (2.9) |
| *General disorders and administration site conditions* | | |
| Injection site pain | 5 (4.9) | 16 (15.5) |
| *Nervous system disorders* | | |
| Akathisia | 3 (2.9) | 2 (1.9) |
| Extrapyramidal disorder | 3 (2.9) | 0 (0) |
| *Cardiac disorders* | | |
| Sinus bradycardia | 4 (3.9) | 2 (1.9) |
| Sinus tachycardia | 0 (0) | 1 (1.0) |
| *Metabolism and nutrition disorders* | | |
| Hyperlipidaemia | 0 (0) | 1 (1.0) |

Table S4: Mean change in Positive and Negative Syndrome Scale (PANSS) total score at 12, 20 and 24 weeks compared to baseline (per-protocol analysis)

| Time (week after first dose) | MS350mg  Least-square means (95% CI) | AM400mg  Least-square means (95% CI) |
| --- | --- | --- |
| ANCOVA | | |
| 12 weeks | -4.92 (-5.95, -3.89) | -4.93 (-5.94, -3.93) |
| 20 weeks | -6.37 (-7.53, -5.21) | -5.70 (-6.82, -4.57) |
| 24 weeks | -7.11 (-8.35, -5.87) | -6.43 (-7.64, -5.21) |
| MMRM | | |
| 12 weeks | -4.7 (-5.7, -3.6) | -4.9 (-5.9, -3.9) |
| 20 weeks | -6.2 (-7.4, -5.0) | -5.8 (-7.0, -4.7) |
| 24 weeks | -7.0 (-8.3, -5.7) | -6.6 (-7.9, -5.3) |

CI: Confidence interval. ANCOVA: analysis of covariation. MMRM: mixed-effect model for repeated measures.

Table S5: Mean change in Clinical Global Impression-Severity (CGI-S) total score at 12, 20 and 24 weeks compared to baseline (per-protocol analysis)

| Time (week after first dose) | MS350mg  Least-square means (95% CI) | AM400mg  Least-square means (95% CI) |
| --- | --- | --- |
| ANCOVA | | |
| 12 weeks | -0.32 (-0.23, -0.13) | -0.23 (-0.14, -0.04) |
| 20 weeks | -0.37 (-0.25, -0.14) | -0.28 (-0.17, -0.06) |
| 24 weeks | -0.42 (-0.30, -0.19) | -0.34 (-0.22, -0.10) |
| MMRM | | |
| 12 weeks | -0.20 (-0.30, -0.11) | -0.13 (-0.23, -0.04) |
| 20 weeks | -0.22 (-0.34, -0.11) | -0.20 (-0.31, -0.08) |
| 24 weeks | -0.26 (-0.38, -0.13) | -0.22 (-0.34, -0.09) |

CI: Confidence interval. ANCOVA: analysis of covariation. MMRM: mixed-effect model for repeated measures.
